# Peer Support in the Management of Diabetes to Improve Cardiovascular Disease Outcomes in Low- and Middle-Income Countries (LMICs)

**DOI:** 10.5334/gh.1263

**Published:** 2023-09-15

**Authors:** Gina Ferrari, Gedeon Ngoga, Aime Manzi, Apoorva Gomber

**Affiliations:** 1 Center for Integration Science, Division of Global Health Equity, Department of Medicine, Brigham and Women’s Hospital, Boston, Massachusetts, USA; 2 Partners In Health (PIH), Rwanda; 3 Kibuye Referral Hospital, Rwanda

**Keywords:** peer support, type 1 diabetes, type 2 diabetes, cardiometabolic disease, cardiovascular disease, LMICs

## Abstract

Diabetes and cardiovascular disease (CVD) contribute to significant morbidity and mortality in low-resource settings. Living with diabetes can be overwhelming, isolating, and exhausting, even in settings of resource availability and health care access, while the psychosocial burden of living with diabetes and CVD can be exacerbated by an increased burden of social determinants of health in low-resource settings. Diabetes and CVD care heavily rely on self-management, and health care professionals are now recognizing the importance of peer support in supporting healthy behaviors, psychosocial well-being, and improved clinical outcomes. However, there is currently a lack of consistency in how peer support programs are defined, implemented, and evaluated.

## Why Is There a Need for Peer Support and What Has Been Done in LMICs?

Noncommunicable diseases (NCDs) are increasingly being viewed as a significant health concern globally, accounting for 74% of global deaths, 86% of which occur in low- and middle-income countries (LMICs) [1]. Cardiometabolic diseases such as heart failure, stroke, and diabetes account for the majority of NCD burden while sharing common risk factors and social determinants of health such as obesity, hypertension, inadequate diet options, and physical inactivity [2]. The *Lancet* Commission on Diabetes report published in 2020 reveals that people in LMICs face a disproportionate burden of the disease, with 80% of cases occurring in these regions [3]. The link between diabetes and cardiovascular disease (CVD) is well defined; diabetes leads to a two to three times increased risk of CVD, and 30% of people living with diabetes (PLWD) die from CVD [4]. Achievement of glycemic targets for diabetes itself can mitigate the risk of poor cardiometabolic outcomes [3], yet most health care teams providing care for PLWD are not meeting recommended targets—less than 25% of people in a sample of 28 LMICs were meeting global diabetes targets of an HbA1c less than 8% [5].

Diabetes requires daily complex medical decisions related to adjusting food intake, physical activity, and medications to maintain blood glucose levels in a desired target range. The psychosocial burden of living with diabetes is well defined in high-resource settings [6], and the body of literature showing similar challenges in LMICs is growing [7,8,9]. Knowledge about the diagnosis and management of diabetes is often low, and dangerous myths persist. Many PLWD face stigma impacting mental health and their ability to perform self-management activities [10,11]. The burden of social determinants of health such as high rates of food insecurity, limited literacy and numeracy, shortages of medications and supplies, and long distances to reach health facilities can exacerbate the psychosocial stressors on PLWD and their families. In LMICs, where provider time is often limited and mental health and behavioral resources may be scarce, community engagement and connection with other PLWD may be the only available resource for addressing these challenges.

The World Heart Foundation’s *Roadmap on the Prevention of Cardiovascular Disease among People Living with Diabetes* [12] defines PLWD as one of three key pillars to promote an integrated approach to improving care for PLWD and CVD. Peer support is an important aspect of this engagement through linking PLWD or CVDs who share similar experiences and identities informed by the direct lived experience of their chronic condition. Research conducted in high-resource settings has shown that peer support interventions for PLWD can improve glycemic outcomes [13] and effectively promote positive health behaviors across diverse cultural settings and groups that suffer from racial, social, or economic injustices [14,15,16,17,18]. However, there is still a lack of understanding about what peer support is and how to implement it in low-resource settings, leading to variability in the programming and monitoring of programs. The extent to which peer support can be optimized by different interventions remains unclear.

In this issue of the *Journal of the American College of Cardiology*, Sherifali et al. report results from a scoping review of peer support interventions for PLWD in LMICs. Inclusion criteria included participants with type 2 diabetes, another CVD, or risk factors for developing CVD and a quantitative study design. Peer support could be delivered in any format (in-person, virtual, phone, web) and any setting (individual, group), as long as it was delivered by a layperson, not a medical professional. Publications were compared with respect to Evan’s definition of peer support, which defines five core functions of peer support, including ‘being there’, ‘assistance in daily management’, ‘social and emotional support’, ‘linkages to clinical care and community resources’, and ‘ongoing support’ [19]. Of the 29 publications that met the inclusion criteria, only 22% were developed in Africa. Given that sub-Saharan Africa (SSA) is expected to experience the largest growth of PLWD (134% increase by 2045) [20], more representative primary research may be needed in these settings. The inclusion of gray literature, program reports, and qualitative research in future scoping reviews may more accurately capture the current state of peer support programs, especially in LMICs where capacity for formal research may be limited.

Several studies evaluated in the scoping review utilized community health workers rather than PLWD or CVDs to implement peer support. As the authors note, the implication and value of the provision of peer support by a member of the community not living with a similar NCD is unclear. Further research may be necessary to differentiate between the roles of a community health worker or peer supporter and the relative value of both. An additional group who may be valuable in the peer support process includes PLWD or CVD who are also health care providers. Though small, this group has the potential to be extremely impactful in their community through integration of medical knowledge in their existing peer community. Peer support programs may also provide valuable space for health care providers not living with an NCD to learn about the lived experience, challenges and practical tools and tricks from daily experiences. Utilization of lay community workers not living with an NCD may blur the lines between peer and community/social support, while exclusion of health care providers from the peer support process may lead to missed learning opportunities.

Use of a broad definition of peer support adds to the strength of this study, given that the needs and structure of peer support programs can vary widely depending on cultural context. Sherifali et al. found that peer support was generally associated with improvement in clinical outcomes such as HbA1c. However, the results highlight a disconnect between the stated goals of peer support programs and how outcomes are currently measured—only 61% of publications reviewed included behavioral or emotional outcomes, while only one looked at the ‘being there’ aspect of peer support. The concept of ‘being there’ along with behavioral and emotional support are arguably the most critical aspects of peer support, yet they were rarely measured in study outcomes. While these outcomes may have been addressed in qualitative studies not evaluated in this review, there is also a need for more structured and standardized formats to quantitatively evaluate behavioral and emotional outcomes related to diabetes and CVD management.

## Future Directions: Expanding Scope, Clearer Outcomes, and Strengthening Integration

The importance of inclusion of the voices of those with different NCDs in all levels of care is increasingly being acknowledged in global guidelines, and expanded availability of peer support will be critical to this process [21]. To date, peer support programs for diabetes in LMICs have been diverse in terms of who implements the support (PLWD, other community members) and how support is delivered (face to face, through text or phone, group, individual) and in the duration of support. However, there are several aspects of peer support that remain poorly understood. One significant aspect of peer support not yet thoroughly described in the literature, especially in LMICs, is mutual aid. Mutual aid includes sharing of food, supplies, and/or medication among PLWD in similar situations, which can be lifesaving in settings of resource scarcity.

Another significant area of peer support not yet addressed in the literature is the use of social media and online communities. Today, more and more people are turning to these communities for support in making informed decisions about their health. However, the impact of social media and online diabetes communities on health outcomes is still not fully understood. Potential benefits that come with the ease of connecting virtually with the diabetes community need to be weighed against known risks associated with social media and internet use, including the spread of medical misinformation and stigma.

While research on peer support in high-resource settings has largely focused on PLWD, Sherifali et al.’s scoping review highlights the value of a more integrated approach to peer support that acknowledges the dual burden of diabetes and CVD and is inclusive of CVD in addition to diabetes. From a health systems perspective, integrated approaches to NCD care have been shown to improve outcomes in LMICs. Promising models such as the Lancet NCDI Poverty Commission’s PEN-Plus program, which has been adopted by WHO/AFRO to improve access to care for PLWD and CVD in rural SSA, has great potential in strengthening multidisease peer support efforts for severe chronic NCDs at intermediate care facilities in LMICs [22,23].

**Figure 1:**
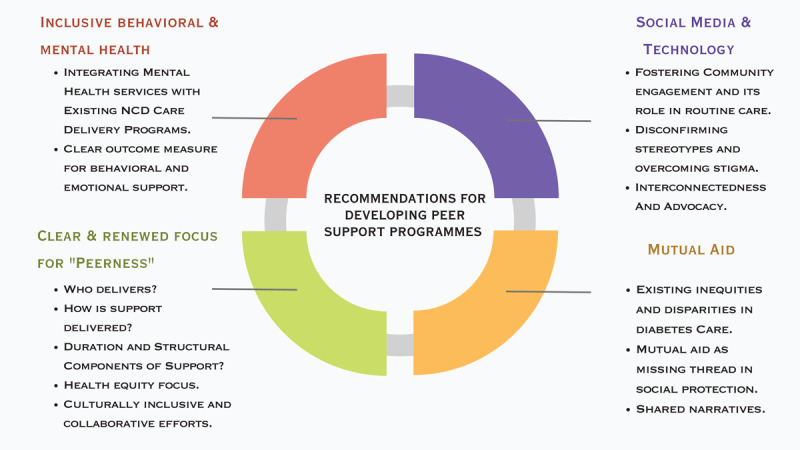
Recommendations for developing diabetes and CVD peer support programs in LMICs.

While broad population-level campaigns are necessary to address the social risk factors that contribute to the development of diabetes and CVD, the estimated projection of 1.3 billion PLWD by 2050 also need improved support. Future studies should focus on defining evidence-based guidelines and programmatic monitoring and evaluation of peer support that is inclusive of emotional and behavioral outcomes. Further exploration of practical ways to enhance peer support for PLWD should be done in collaboration with those living with diabetes and CVD and should focus on cultural perceptions, increasing representation, and prioritizing the diverse needs of those in LMICs (Figure 1).
